# A paradigm shift in simulating affinity maturation to elicit broadly neutralizing antibodies

**DOI:** 10.3389/fimmu.2025.1627674

**Published:** 2025-07-01

**Authors:** Fahsai Nakarin, Kayla G. Sprenger

**Affiliations:** ^1^ Biomedical Engineering Graduate Program, University of Colorado Boulder, Boulder, CO, United States; ^2^ Department of Chemical and Biological Engineering, University of Colorado Boulder, Boulder, CO, United States

**Keywords:** affinity maturation, germinal center, simulation, broadly neutralizing antibody, B cell

## Abstract

Broadly neutralizing antibodies (bnAbs) offer a promising route to protect against rapidly evolving pathogens such as HIV, influenza, and SARS-CoV-2, yet eliciting them through vaccination remains a significant challenge. A key to this problem lies in understanding antibody affinity maturation (AM), the evolutionary process within germinal centers (GCs) that shapes the B cell and thus antibody response. Traditionally, AM has been viewed as favoring the selection of B cells with the highest-affinity B cell receptors (BCRs) through competitive interplays. However, emerging evidence suggests that GCs are more permissive, allowing B cells with a broad range of affinities to persist, thereby promoting clonal diversity and enabling the rare emergence of bnAbs. This review reassesses affinity-based selection models and proposes a new paradigm that integrates multifactorial processes, including stochastic B cell decisions within GC dynamics, antigen extraction efficiency influenced by probabilistic bond rupture, and avidity-driven BCR binding alterations and representations on multivalent antigens. We highlight how advanced AM simulations that move beyond affinity as the sole determinant provide a more realistic and predictive representation of AM, marking a major step forward in developing strategies to promote effective immune responses against highly mutable, complex antigens.

## 1 Introduction

Antibody affinity maturation (AM) is a dynamic evolutionary process orchestrated primarily within germinal centers (GCs) (1–3), where antibody-producing B cells undergo rounds of somatic hypermutation (SHM) and selection. During SHM, mutations are introduced into antibody genes as B cells proliferate, followed by a competitive selection mechanism that favors B cells with enhanced antigen-binding affinity. This iterative cycle of mutation and selection drives the generation of competent antibodies essential for effective immune responses (4).

The predominant principle of AM involves a stringent process aimed at generating antibodies with high antigen affinities (5). However, this understanding is challenged by the spectrum of antibody affinities observed experimentally (6–10). Permissive GCs allow low-affinity B cells to mature and diversify the clonal population (9, 10), a strategy that supports the generation of broadly neutralizing antibodies (bnAbs), which prioritize breadth over depth (11). Higher affinities do not always correlate with improved competence when dealing with highly mutable complex antigens (12, 13), instead, the ability to recognize a wide range of antigen variants, as seen with bnAbs, appears more effective (14). This prompts a reevaluation of the affinity-based discrimination theory and whether binding affinity should remain the sole measure of antibody maturation.

Understanding how GCs balance stringency and permissiveness during AM is critical for informing vaccine strategies aimed at eliciting bnAbs. However, experimental access to GC dynamics is limited. Simulations provide an unrestricted theory-testing space to derive novel predictions of permissive GC responses promoting the rare emergence of bnAbs (15, 16). In recent decades, computer-aided simulation tools have emerged, leveraging experimental findings and mathematical algorithms to model the complex cellular interactions and selection pressures within GCs (17), thus guiding the iterative AM process to tailor antibody characteristics such as high breadth (18–22). The accuracy and translatability of *in silico* findings depend on models that align with essential biological principles. A single theory that completely explains the evolutionary dynamics of AM under GC responses does not yet exist, and some mechanisms of GC reactions remain elusive.

Building on the foundational overview by Buchauer and Wardemann in 2019 (17), we explore recent strategies for modeling AM, with a specific focus on incorporating crucial features of GCs (3) that may influence the emergence of bnAbs. In particular, we address three central questions: (1) What biological mechanisms promote diversity during AM within GCs? (2) How are these mechanisms represented in current computational models? (3) What emerging modeling techniques offer new opportunities to advance AM simulations in support of guiding immune responses against highly mutable pathogens? This review synthesizes updated insights into GC dynamics and emerging trends in computational techniques, offering a framework to pursue new paradigms in AM modeling.

## 2 Recapping germinal center dynamics

**Figure 1:**
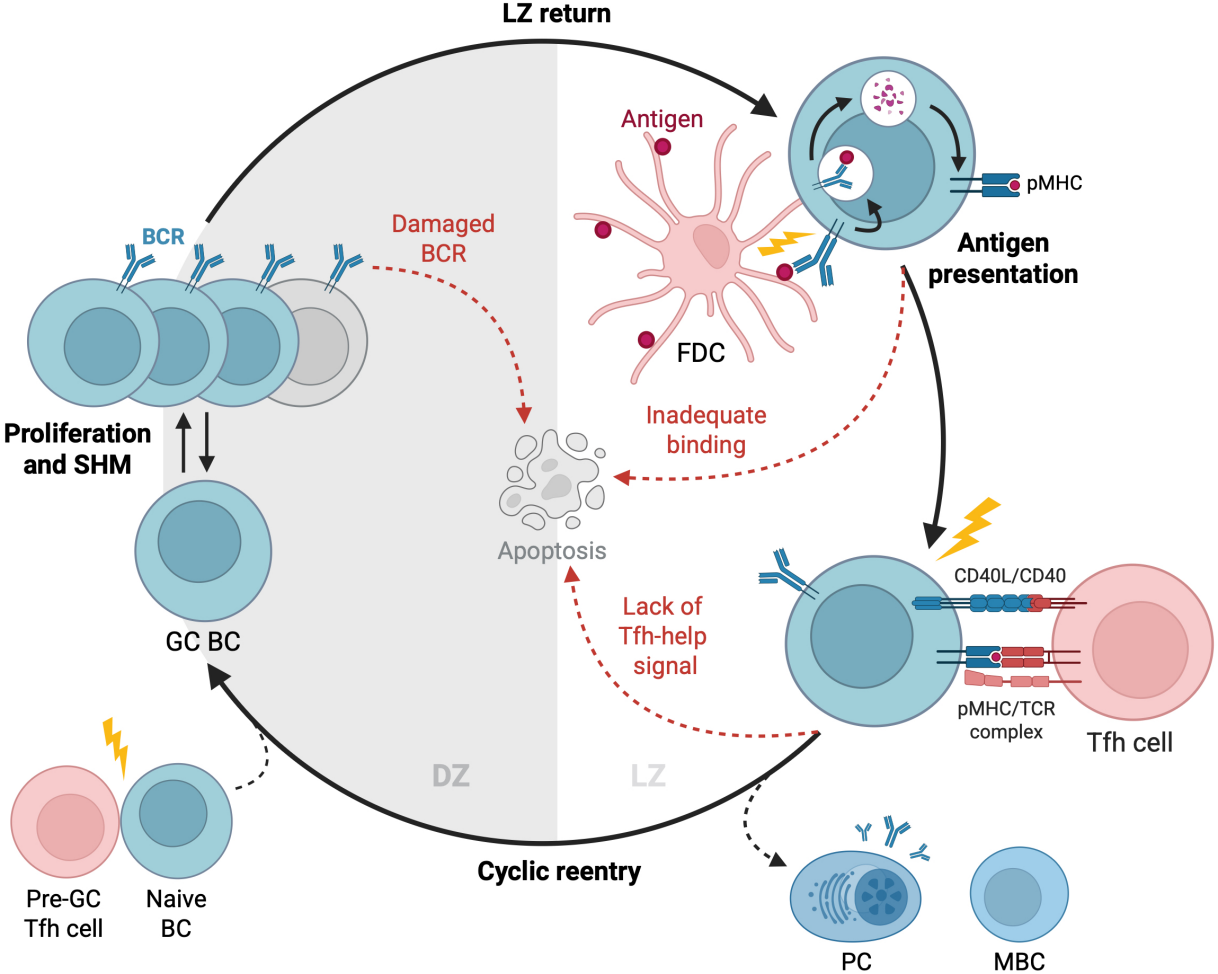
Dynamics of GC reactions. Activated B cells enter the GC and undergo cycles of proliferation, SHM, and selection to improve their antigen affinity. In the dark zone, B cells proliferate and mutate before migrating to the light zone to test their BCRs against antigens presented by FDCs. B cells that successfully bind antigens and receive signals from Tfh cells can re-enter the dark zone for further rounds of division and BCR mutation or exit the GC to differentiate into antibody-secreting PCs or MBCs. B cells that fail to bind antigens, presumably not receiving these signals, undergo their default apoptosis. GC, Germinal Center; BC, B Cell; DZ, Dark Zone; SHM, Somatic Hypermutation; FDC, Follicular Dendritic Cell; LZ, Light Zone; PC, Plasma Cell; MBC, Memory B Cell. Created with https://BioRender.com.

GCs are dynamic microenvironments where B cells, T follicular helper (Tfh) cells, and follicular dendritic cells (FDCs) engage in complex interactions driving antibody evolution (**Figure 1**). Upon formation by activated B cells after infection or immunization, GCs exhibit a distinct spatial organization with two main regions: the dark zone and the light zone (1). The dark zone is a site of rapid B cell proliferation and SHM, while the light zone is where B cells undergo affinity-based selection and receive help from Tfh cells (2, 23, 24). Notably, most B cells degrade their pre-SHM B cell receptors (BCRs) before exiting the dark zone, and those bearing dysfunctional BCRs due to SHM undergo apoptosis at this stage. This checkpoint ensures that only B cells with functional, somatically mutated BCRs proceed to the light zone for selection (25). In the light zone, FDCs display antigens on their surface, allowing B cells to test the affinity of their receptors for the antigen. Higher-affinity B cells collect more antigens from FDCs, leading to a higher density of antigen-derived, peptide-loaded histocompatibility complexes (pMHC) on their surface that can be recognized by the limited number of Tfh cells (26, 27). This interaction facilitates the death-limited selection (i.e., Tfh-cell selective survival license) of B cells with higher affinity receptors to re-enter the dark zone for further rounds of proliferation and mutation (28, 29), while those with lower affinity undergo apoptosis due to neglect (2, 5).

While the GC mechanism generally favors higher-affinity B cells, the process is not entirely stringent, allowing lower-affinity clones to persist—an observation not fully explained by the death-limited model. In this model, help from Tfh cells is a crucial determinant of B cell survival (5, 30). A remarkable work by Bannard and colleagues demonstrated that T-cell help is not required to initiate cyclic reentry into the dark zone (31). Instead, pMHC-dependent interactions with Tfh cells gradually refuel B cells, aiding their survival in the dark zone through prolonged dwell times and accelerated cell cycles, increasing the likelihood of reentry into the light zone (31–33). Unlike the death-limited theory, the birth-limited selection model proposes that a B cell’s ability to proliferate after re-entering the dark zone depends on the strength of signals received in the light zone (3). This model aligns with the aforementioned findings by Bannard and colleagues and allows for a broader range of affinities to be selected, as B cells are not strictly eliminated based on affinity but are given varying opportunities to proliferate (34, 35). Consequently, overall diversity is maintained by allowing lower-affinity clones to persist until clonal bursts occur, where a single B cell clone rapidly expands (6). Although the cyclic re-entry initiation mechanism remains under investigation, the evidence presented above suggests that the deterministic, death-limited selection model, which relies solely on Tfh cell help and a constant number of B cell divisions, may need revision.

An alternative approach to understanding the selection process in GCs involves integrating GC models with intracellular molecular networks to manipulate AM, specifically through the upregulation of cell-cycle regulators such as the transcription factor c-Myc (7). The induction of c-Myc is regulated by a combination of BCR signaling and Tfh cell-derived signals (36). BCR engagement primes B cells to receive help from Tfh cells (37), which provide additional signals like CD40 ligation and cytokines, fully activating c-Myc expression. This expression is induced in a small subset of light zone B cells associated with positive selection (38), marking them for further proliferation (39). Meyer-Hermann introduced a simulation framework incorporating these molecular networks, accounting for both Tfh cell-dependent and independent pathways, thus allowing for separate control of selection and division (40). This framework predicts varying outcomes for high- and low-affinity B cells, such as differences in light zone passage times and division numbers, which were validated experimentally (31). However, the precise transcriptional regulatory networks governing the positive selection of B cells remain incompletely understood. Rather than relying on explicit molecular mechanisms, models incorporating coarse-grained versions of antagonistic regulators between BCR signaling and Tfh-cell have been developed to replicate biologically relevant dynamics in GCs without predefining deterministic, affinity-based decisions (41). Similarly, Martínez’s research group developed a probabilistic model of GC reactions inspired by the stochastic kinetics of cellular reactants (42), demonstrating that clonal diversity is reduced over time due to clonal dominance driven by division bursts arising from slight stochastic advantages in antigen affinity (43).

Beyond cyclic reentry, B cells can exit GCs by differentiating into antibody-secreting plasma cells (PCs) or long-lived memory B cells (MBCs), though the mechanisms underlying this fate decision remain unclear. Some studies suggest that B cell signaling mediated by Tfh cells influences this decision in the light zone, with high-affinity B cells more likely to differentiate into PCs (44–46), while lower-affinity B cells tend to become MBCs (47, 48). Other studies propose that antigens are asymmetrically distributed during cell division, leading to antigen-retaining daughter cells differentiating into either PCs or MBCs based on the cellular signals they acquire and exit the GC through the dark zone, while those without antigens remain engaged in the GC reaction (5, 49, 50). Additionally, evidence supports a temporal switch (51) in GC reactions, where PC differentiation is favored in later GC stages and MBCs are primarily derived from earlier GC stages. However, high-affinity pre-MBCs have also been identified in late-stage GCs, supported by the observation that high-affinity antibodies with extensive SHM are commonly derived from the human MBC pool (3, 52). Recent findings by Sprumont et al. (9) and Sutton and Gao et al. (10) challenge this view, showing PCs can emerge at any stage of the GC reaction, independent of BCR affinity or temporal pattern during the GC reaction (e.g., early versus late stages). Taken together, these findings suggest that our understanding of the criteria guiding the fate determination of B cells is not yet absolute. Where the underlying mechanisms are less understood, stochastic, probabilistic models provide an alternative to address the complexity and variability in GC dynamics. By incorporating stochastic interactions and decision-making guided by cellular and molecular machinery, simulations can better align with experimental data (41, 43, 53–55).

## 3 Reconsidering antigen collection mechanisms

A key survival strategy for B cells involves their recognition by Tfh cells, mediated by the presentation of antigens on the B cell surface, which reflects the amount of antigen acquired from FDCs (27). Antigen capture occurs through physical extraction via BCRs, with the efficiency of this process driven by the BCR-antigen interaction. Stronger-binding BCRs are better able to withstand the mechanical pulling forces exerted by B cells (56, 57). The assumption that higher-affinity B cells lead to enhanced interactions with Tfh cells holds true for simple antigens with a single epitope, like haptens (58), where BCR affinities directly correlate with the number of antigens presented, driving the purifying selection of the most competent clones. However, the model becomes more nuanced for antigens with multiple epitopes (59, 60), where permissive selection allows the survival of the less-fit clones concerning their affinity against a pre-defined epitope. This survival suggests that the definition of strong-binding BCRs depends on more than single-epitope affinity, and other metrics such as probabilistic bond rupture (61, 62), avidity (63), and bivalent effects (18) can redefine what constitutes strong binders. Incorporating additional factors beyond affinity provides an alternative framework for recapitulating the dynamics of antigen collection mechanisms, thus, reproducing permissive selection processes in GCs.

**Figure 2:**
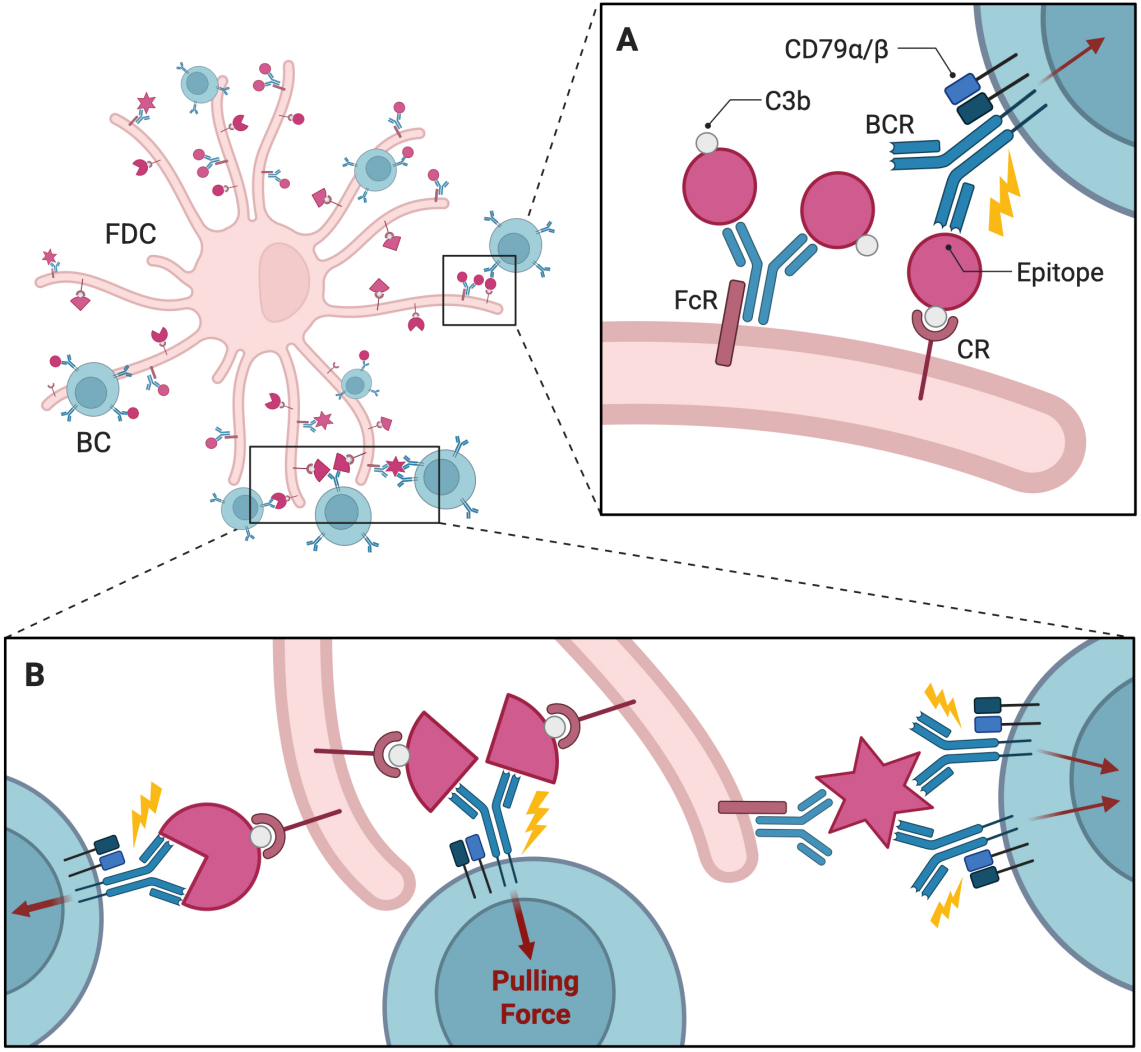
Interactions between FDC and B cells and in the GC. The FDC displays antigens on its surface, often in the form of immune complexes bound to antibodies and complement proteins like C3b. **(A)** Fc or complement receptor secures the immune complexes on FDC, while BCRs bind to specific epitopes on the tethered antigen. BCR-antigen interactions trigger BCR signaling through associated molecules like CD79α/β, leading to downstream processes such as antigen internalization. **(B)** The mechanical pulling force exerted by B cells to extract antigens from the FDC is crucial for efficient internalization and processing of the antigen. The strength of this pulling force depends on how tightly the BCR interacts with the antigen’s epitopes. FDC, Follicular Dendritic Cell; BC, B Cell; GC, Germinal Center; CR, Complement Receptor; FcR, Fc Receptor; BCR, B Cell Receptor. Created with https://BioRender.com.

Evidence from many studies suggests the existence of an affinity ceiling *in vivo*, potentially determined by the strength of antigen tethers (64), Fc receptors, or complement receptors on the FDC membrane (**Figure 2A**). This implies that exceptionally high-affinity BCRs gain little advantage over moderate-affinity BCRs in GCs. In contrast to traditional equilibrium-based models, where binding affinity depends on static association and dissociation rates, Jiang and Wang developed a theoretical framework integrating nonequilibrium mechanical forces exerted by B cells and the physical properties of antigen tethers (61) In their model, the success of antigen extraction depends on the magnitude and duration of the applied force relative to the tether strength. If the applied force exceeds the tether strength, the antigen is extracted, influencing clonal selection. The model predicts a limit to how much force can be applied before reaching the affinity ceiling, beyond which further increases in affinity do not significantly enhance antigen extraction. Another alternative to affinity-based antigen collection is the probabilistic rupture mechanism proposed by Lashgari et al. Here, bonds between BCRs and antigens break with a probability dependent on the speed of binding (association) and unbinding (dissociation) (62). B cells with slower dissociation rates outcompete those with faster association rates because bonds with slower dissociation rates are less likely to rupture during extraction attempts, translating into more successful antigen collection.

Affinity refers to the strength of a single interaction between a BCR and its epitope, while avidity describes the cumulative strength of multiple interactions in polyvalent systems where multiple epitopes are presented. A BCR has two arms capable of engaging in both monovalent (single interaction) and bivalent (dual interaction) binding modes (**Figure 2B**), and multiple BCRs can simultaneously interact with polyvalent antigens, increasing overall binding strength through avidity (65). This enhancement is observed even when the affinity of individual BCR-antigen interactions is relatively weak (63, 66). Building on the assumption of monovalent BCR-antigen interactions, a coarse-grained AM model from Ovchinnikov and Karplus incorporates both monovalent and bivalent binding modes to explore differences in avidity based on binding valency (63). In this model, the interaction between BCRs and antigens is expressed by two equilibrium constants, corresponding to the first and second BCR arm, which vary depending on the strategy each clone employs the second arm to engage in binding. In their simulations, cooperative bivalent binders (i.e., stronger binding after the first arm engages) outcompeted non-cooperative bivalent and monovalent binders, achieving greater AM and memory cell production. Amitai et al. developed a complementary framework for bivalent binding, considering epitope accessibility with respect to antigen geometry (18). Using molecular dynamics simulations, they calculated the on-rate of BCR binding for the first and second arms based on the favorable geometry of nearby epitopes. These rates are crucial for efficient antigen capture and subsequent B cell activation in their GC simulations. Notably, consistent with the previously discussed coarse-grained model developed by Ovchinnikov et al., avidity-enhanced bivalent binding plays a key role in determining immunodominance hierarchies by preferentially selecting epitopes that enable such interactions. It is also worth noting that vaccine design is often targeted towards subdominant but protective epitopes, such as those recognized by bnAbs. Bivalent binding is thought to reproduce the competitive pressures in GCs that select for clones targeting these subdominant epitopes.

While BCR affinity, mediated by the complementarity-determining regions (CDRs), is critical for antigen recognition through epitope binding bnAbs often contain a high number of mutations in the framework regions (FWRs), which play a key role in governing the flexibility (67, 68) necessary for bivalent binding and broad antigen recognition (18). More flexible antibodies experience greater entropy loss upon binding, making their binding less favorable thermodynamically compared to rigid antibodies. However, the flexibility alternatively offers an advantage, as it enables them to bind a broader array of antigen variants by adopting multiple conformations (68). Flexibility parameters can be incorporated into AM modeling to influence the CDR-directed binding free energy, as they affect the structural rigidity or flexibility toward conserved and variable regions (69). The AM model that incorporates the flexibility parameter predicts a distinct evolutionary pathway for BCRs with low to moderate affinity for conserved epitopes. Initially, FWR mutations increase flexibility, allowing antibodies to bind a broader range of antigen variants. Over time, however, antibodies evolve toward greater rigidity, enhancing specificity and potency for conserved epitopes.

## 4 Reconstructing antibody-antigen representations

In the context of simulating AM, the selection of B cells is typically guided by energy functions derived from the interactions between BCRs/antibodies and antigens (17). These energy functions primarily quantify the binding affinity between antibodies and antigens, which is crucial for accurately modeling the evolution of BCRs towards matured antibodies. The quality of such simulations tied to energy functions, particularly when dealing with complex antigens, depends significantly on how well the antibody-antigen representations capture the intricacies of epitope immunodominance (12, 16). This phenomenon refers to the preferential targeting of specific epitopes during immune responses, influenced by factors such as epitope accessibility, antigen valency, and glycosylation patterns. For instance, epitopes that are more accessible or structurally exposed are more likely to dominate the immune response, while those that are concealed or masked by glycosylation are often overlooked. Additionally, antigen valency—the number of epitopes presenting on an antigen—can influence the number of BCRs that can bind simultaneously, thereby affecting the binding strength and downstream processes (discussed in Section 3). These factors should be considered in antibody-antigen representations to ensure the accuracy of the simulations.

Sequence-based approaches are computationally efficient for large-scale studies. However, their implementation requires careful consideration to effectively represent the complexity of multi-epitope interactions with varying degrees of accessibility in the shape space, as thoroughly discussed in a review by Robert and colleagues (16). To address this complexity, when epitopes are well-characterized, some studies suggest treating each antigenic site as an independent entity (70, 71). This strategy allows the simulations to capture variations in immune responses by accounting for differences in how antibodies bind to distinct regions of the antigen, reflecting the phenomenon of epitope immunodominance. However, when the knowledge of epitopes is not well understood, introducing weighted parameters or penalties into the calculations can help mitigate interactions with hidden or variable regions (19, 72). This approach provides a coarse-grained yet functional representation of the complex antigenic landscape. By adjusting these weights, the model can simulate how certain epitopes may be less accessible or exhibit greater variability, thus influencing patterns of immunodominance and cross-reactivity, the latter of which is a multifaceted property (16). It can refer to polyreactivity, where structural mimicry drives the recognition of different variants, promiscuity, where sequence similarity leads to broad recognition, or conserved site recognition, where antibodies target conserved regions across different strains. Sequence similarity metrics and the adjustable weights are particularly useful for assessing cross-reactivity related to conserved site recognition or promiscuity. However, these metrics are limited in their ability to fully capture the nuanced interactions involved in polyreactivity, where structural similarities may not be solely determined by sequence. On the other hand, structure-based representations are less sensitive to sequence-level dissimilarities. For example, the lattice-based representation model by Robert et al. allows for the modeling of multiple possible binding conformations, each with minimized energy states (73). This flexibility, although computationally more expensive, makes structural approaches better suited for capturing the complexity of both epitope accessibility and cross-reactivity of multivalent antigens. By incorporating structural representations of BCR-antigen interactions, the model can capture the permissive nature of GCs (74), which is primarily driven by the antigen with the highest immunogenicity but also remains permissive to antigens with lower immunogenicity.

Many promising strategies have emerged from the coarse-graining of antibody-antigen interactions and antibody evolution, with coarse-grained models becoming the standard approach for simulating AM over several decades (16, 17). Despite their versatility and wide applicability, coarse-grained models overlook critical molecular details of the evolving antibodies in relation to specific epitopes, which can now be captured by the high-resolution atomic AM models (75). In contrast to the coarse-grained models, which typically use shape space representations and assume uniformly distributed mutations, this high-resolution model integrates experimentally observed biases in mutational patterns mediated by the activation-induced cytidine deaminase (AID) enzyme (76). This implementation enables more accurate simulations of SHM in B cells and allows for the reconstruction of evolutionary trajectories, tracing the journey from a germline BCR sequence to the reactive progeny of antibodies targeting a structured epitope at a nucleotide level. While glycosylation patterns and some less common mutations, such as insertions and deletions, remain beyond the current capabilities of the models, the evolutionary insights provided by the high-resolution models hold the potential to significantly enhance our understanding of antibody evolution and immune response. For instance, they could estimate the time required for the emergence of bnAbs, identify convergent solutions arising from different B cell precursors, and personalize immunization plans.

## 5 Discussion

AM simulations have been programmed based on the premise that competition-driven selection and fate decisions depend primarily on affinity-mediated Tfh cell help. However, emerging studies challenge this view, revealing that GCs exhibit permissive properties, both with and without Tfh cells, that allow high-affinity B cells to thrive while preserving clonal diversity. Rather than relying solely on deterministic Tfh-cell-dependent selection, manipulating B cell proliferation and fate through stochastic cellular and molecular interactions appears to better account for the maintenance of this diversity. The concept of an affinity ceiling further suggests that B cell clones do not always outperform others with their higher-affinity BCRs due to the intrinsic FDC-antigen tether strength. The additional mechanisms, besides affinity, to confront antigens underscore the permissive nature of the GC, where selection is not governed solely by the strength of a single BCR-antigen interaction but by a broader set of criteria, including the dynamic bond rupture and the flexibility of BCR binding. Nevertheless, the simulations depend on sophisticated and interpretable models that require accurate representations of the complex interactions between BCRs and antigens. By incorporating these multifactorial influences, AM simulations could offer a more nuanced understanding of the antibody evolution that goes beyond affinity alone.

While this review primarily focuses on modeling single-GC dynamics and intra-GC selection (see **Table 1** for a summary), it is important to recognize that inter-GC communication and antibody-mediated feedback—such as epitope masking and selection bias from pre-existing antibodies—play crucial roles in shaping the broader AM landscape. Several experimental and computational studies have demonstrated how these feedback mechanisms can bias the recall repertoire away from the original immunogen and inhibit the recruitment of B cells with similar specificity into secondary GCs, thereby affecting the potential for breadth (72, 77–79). Although these multi-GC and feedback-driven mechanisms are outside the scope of this review, they represent essential elements of the humoral immune response that should be incorporated into AM simulation frameworks aiming to model vaccination scenarios and optimize bnAb elicitation.

**Table 1:** Summary of selected affinity maturation models.

Topic	Article	Contribution	Interesting Feature
Understanding Molecular mechanisms	Pélissier et al., 2020 (42, 43)	A loss of clonal diversity from clonal dominance is a result of clonal bursts	The cellular interactions of B, Tfh cells and FDCs were modelled stochastically.
	Meyer-Hermann, 2021 (40)	Revision of re-cyclic decision model	Intracellular molecular networks determine B cell fates.
	Yan et al., 2022 (41, 53)	Introduction of bystander effects	Cellular and molecular networks drive stochastic B cell decisions.
Manipulating antibody-antigen interactions	Ovchinnikov et al., 2018 (69)	B cells initially increase flexibility to bind antigen variants and later evolve toward rigidity for specificity.	Effects of FWR mutations on antibody flexibility and antigen binding
	Amitai et al., 2020 (18)	Epitopes that allow for bivalent interactions are favored by B cells.	A bivalent effect influences antigen collection.
	Lashgari et al., 2022 (62)	The kinetic selection mechanism benefits clones with lower dissociation rates.	A probabilistic bond rupture inters dissociation rates of BCR-antigen contacts.
	Ovchinnikov & Karplus, 2022 (63)	The GC is easily dominated by B cells with cooperative bivalent binding.	Affinity is re-defined in a term of avidity.
	Jiang & Wang, 2023 (61)	B cells are selected based on ability to engage with and extract antigens.	Extraction probability is based on the kinetics of bond rupture.
Modulating antibody-antigen representations	Anderson et al., 2020 (70, 71)	The model tracks immune responses at distinct antigenic sites, showing different epitope immunodominance.	Representations of multiple antigenic epitopes in a shape space
	Robert et al., 2021 (73, 74)	The framework allows for multiple binding conformations of antibodies interacting with epitope variants.	3D Structural representations of antigens with surface amino acid compositions and topologies
	Conti et al., 2022 (75)	The simulation captures a full evolutional path at a residue level.	Incorporating the AID-mediated mutational biases during SHM
	Yang et al., 2023 (72)	Subdominant epitope-targeting B cells tend to develop cross-reactivity.	Two-epitope model of dominant and subdominant epitopes
